# Interferon Tau Alleviates Obesity-Induced Adipose Tissue Inflammation and Insulin Resistance by Regulating Macrophage Polarization

**DOI:** 10.1371/journal.pone.0098835

**Published:** 2014-06-06

**Authors:** Wei Ying, Srikanth Kanameni, Cheng-An Chang, Vijayalekshmi Nair, Stephen Safe, Fuller W. Bazer, Beiyan Zhou

**Affiliations:** 1 Department of Animal Science, Texas A&M University, College Station, Texas, United States of America; 2 Department of Veterinary Physiology and Pharmacology, College of Veterinary Medicine & Biomedical Sciences, Texas A&M University, College Station, Texas, United States of America; University Medical Center Freiburg, Germany

## Abstract

Chronic adipose tissue inflammation is a hallmark of obesity-induced insulin resistance and anti-inflammatory agents can benefit patients with obesity-associated syndromes. Currently available type I interferons for therapeutic immunomodulation are accompanied by high cytotoxicity and therefore in this study we have examined anti-inflammatory effects of interferon tau (IFNT), a member of the type I interferon family with low cellular toxicity even at high doses. Using a diet-induced obesity mouse model, we observed enhanced insulin sensitivity in obese mice administered IFNT compared to control mice, which was accompanied by a significant decrease in secretion of proinflammatory cytokines and elevated anti-inflammatory macrophages (M2) in adipose tissue. Further investigations revealed that IFNT is a potent regulator of macrophage activation that favors anti-inflammatory responses as evidenced by activation of associated surface antigens, production of anti-inflammatory cytokines, and activation of selective cell signaling pathways. Thus, our study demonstrates, for the first time, that IFNT can significantly mitigate obesity-associated systemic insulin resistance and tissue inflammation by controlling macrophage polarization, and thus IFNT can be a novel bio-therapeutic agent for treating obesity-associated syndromes and type 2 diabetes.

## Introduction

Obesity and its associated metabolic abnormalities, including insulin resistance and cardiovascular disorders, have reached epidemic proportions. Chronic low degree adipose tissue inflammation, accompanied by enhanced immune cell infiltration, is a hallmark of obesity and a crucial contributor to the pathogenesis of insulin resistance and metabolic diseases [1]–[7]. The infiltrated immune cells play critical roles in modulating obesity-associated adipose tissue inflammation. Among them, macrophages account for up to 50% of the stromal cell population in adipose tissues of obese individuals and are critical regulators of adipose tissue functions [3], [8]–[11]. In addition, adipose tissue macrophages (ATMs) undergo a phenotypic switch from anti-inflammatory status (M2) in the adipose tissues of lean individuals to a proinflammatory (M1) status in adipose tissues of obese subjects, which results in the development of tissue inflammation and systemic insulin resistance [12], [13]. The classic proinflammatory responses of ATMs (M1) depend on Toll-Like Receptors (TLRs) and activation of nuclear factor κB (NFκB)/c-Jun N-terminal kinase (JNK), leading to the production of inflammatory cytokines [14]. In contrast, activation of M2 ATM leads to recruitment of peroxisome proliferator-activated receptor γ (PPARγ) or other transcription factors resulting in an anti-inflammatory status [13], [15]–[17]. Our recent study revealed that other molecules, such as microRNAs, can exhibit profound regulatory functions on macrophage polarization [18]. It has been demonstrated that activation of M2 macrophages can improve systemic insulin sensitivity and protect against development of cardiovascular diseases and type 2 diabetes [19], [20].

Clinical studies show the anti-inflammatory treatments can benefit patients with systemic insulin resistance [21]–[23]. Type I interferons have been used as anti-inflammatory therapies by suppressing production of inflammatory cytokines such as interleukin (IL)-1β and tumor necrosis factor α (TNF-α) [24]–[26].However, severe cell toxicities associated with currently available type I IFNs, such as IFN-α and -β (IFNA and IFNB) [27], [28], dramatically hinder their clinic application. Recent studies revealed that a member of type I IFN family, interferon tau (IFNT), exerts potent immunomodulatory effects with very low cytotoxicity even at high dosages, which providessupport for a clinic application of IFNT.

IFNT is a type I interferon produced exclusively by trophectoderm cells of conceptuses of ruminant species and plays a central role for successful implantation and establishment of pregnancy [29]. IFNT shares high protein structural similarities with other members of the type I interferon family, such as IFNA and IFNB and particularly interferon omega (IFNW) [30]. Compared with IFNA and IFNB that have been applied as clinical therapeutics, IFNT lacks their cytotoxicity even at high concentrations [31], [32]. Previous studies have shown that IFNT can shift the immune profile from an inflammatory to an anti-inflammatory phenotype to mitigate against autoimmune-associated diseases [33], [34]. Here, we show that IFNT is a potent regulator of macrophage polarization that significantly suppresses obesity-associated adipose tissue inflammation and ameliorates systemic insulin resistance. Thus, this study provides important evidence, for the first time, toward understanding the regulatory mechanism of IFNT action in the context of obesity and indicates potential clinical applications for IFNT in treating obesity-associated metabolic syndrome.

## Materials and Methods

### Mice

**Figure 1:**
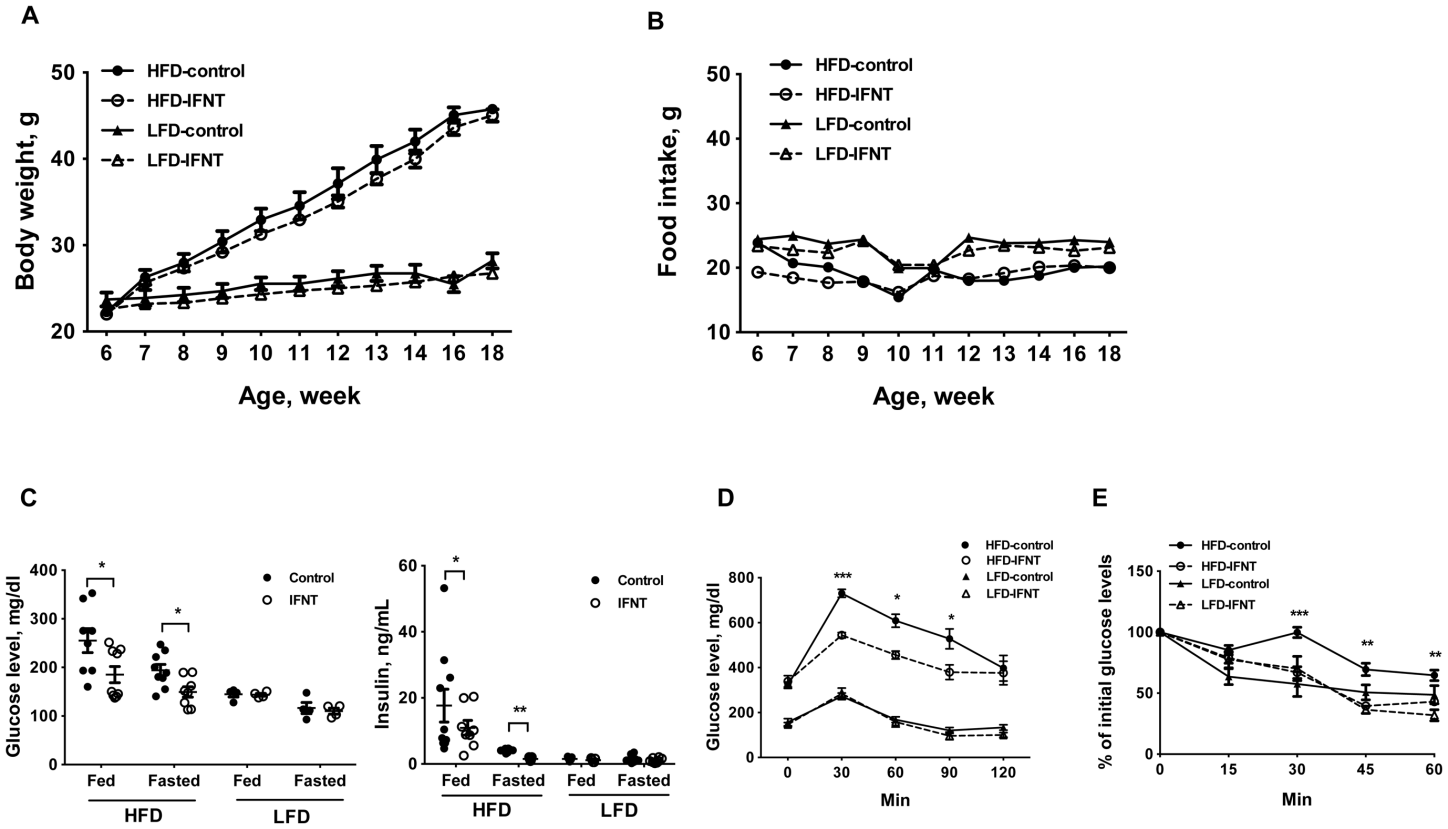
Interferon tau (IFNT) alleviates high-fat diet (HFD)-induced insulin resistance. Body weight (A) and food intake (B) of mice were monitored during a 12-week feeding period (n = 9–10). (C) Concentrations of glucose and insulin in plasma of control or IFNT-treated mice fed a HFD or low-fat diet (LFD), or fasted for 16 h. (D) Glucose tolerance test (n = 6). (E) Insulin tolerance test (n = 6). Data are presented as mean ± SEM. *P<0.05, **P<0.001, ***P<0.0001.

Male C57BL/6J mice 6 weeks of age were used for diet feeding and bone marrow isolation and macrophage activation analyses. All mice were maintained on a 12-/12-hour light-dark cycle and fed *ad libitum*. To induce obesity, mice were fed a high-fat diet (HFD; 60% fat calories, 20% protein calories, and 20% carbohydrate calories; Research Diets, Inc) for 12 weeks. Mice fed on a low-fat diet (LFD; 10% fat calories, 20% protein calories, and 70% carbohydrate calories; Research Diets, Inc) served as controls. In the IFNT treatment, mice received recombinant IFNT via drinking water (8 µg/kg body weight/day) for 12 weeks [21]. The IFNT delivered orally in drinking water was detected in plasma of mice using a highly specific and sensitive radioimmunoassay (FigureS1) [35]. Glucose metabolism and insulin sensitivity of LFD and HFD mice were evaluated by measuring concentrations of glucose and insulin in plasma, andconducting glucose tolerance and insulin tolerance tests. The glucose tolerance and insulin tolerance tests were performed during week 12 of the study, and there was a 6-day interval between these two tests. In addition, the glucose tolerance and insulin tolerance tests were performedbetween 8:00 AM and 10:00 AM for all mice. Blood samples were collected fromthe tail vein in EDTA-coated tubes, and plasma was harvested afterthe blood was centrifuged at 1,000×g for 15 min at 4°C. At the end of the study, all mice were euthanized between 8:00 AM and 10:00 AM by exposure to high dosage of carbon dioxide (CO_2_).In this study, we combined the visceral adipose tissues (VAT; retroperitoneal fat, kidney fat, gastrointestinal fat, epididymal fat) for isolation of mature adipocytes, measurement of metabolic gene expression, immunohistochemistry, and evaluation of immune cell infiltration. The VAT from mice were fixed in fresh 4% paraformaldehyde andused for immunohistochemical analysis or snap frozen in liquid nitrogen and stored in −80°C. All study protocols were approved by the Institutional Animal Care and Use Committee of Texas A&M University.

### Mature adipocyte isolation and *ex vivo* analysis of insulin signaling

VAT wasmincedand digested in Hank's Balanced Salt Solution (HBSS) digestion buffer containing 1 mg/mL of collagenase II, 1% BSA and 100 mM HEPES for 40 min at 37°C.After passing through a 250 µm nylon mesh,the stromal cells and mature adipocytes were separated by centrifuging at 1,500×g for 5 min at 4°C.For *ex vivo* analysis of insulin signaling, the mature adipocytes were collected and stimulated with or without 100 nM insulin in Dulbecco's Modified Eagle Medium/Nutrient F-12 Ham (DMEM/F12)for 15 min, followed by cell lysis in Radio-Immunoprecipitation Assay (RIPA) buffer containinga protease/phosphatase inhibitor cocktail.

### Bone marrow isolation and macrophage differentiation

Bone marrow-derived macrophages (BMDMs) were obtained as described previously [36]. After red blood cell lysis, bone marrow cells were seeded at 2×10^6^ cells/mL with Iscove's Modified Dulbecco's Medium (IMDM) medium containing 10% FBS and 15% L929 culture supernatant as a source of granulocyte macrophage colony-stimulating factor(GM-CSF) for differentiation of bone marrow cells to monocytes. After 7 days, the formation of mature monocytes was evaluated by flow cytometry using antibodies against CD11b and F4/80.

### Macrophage polarization analysis

**Figure 2:**
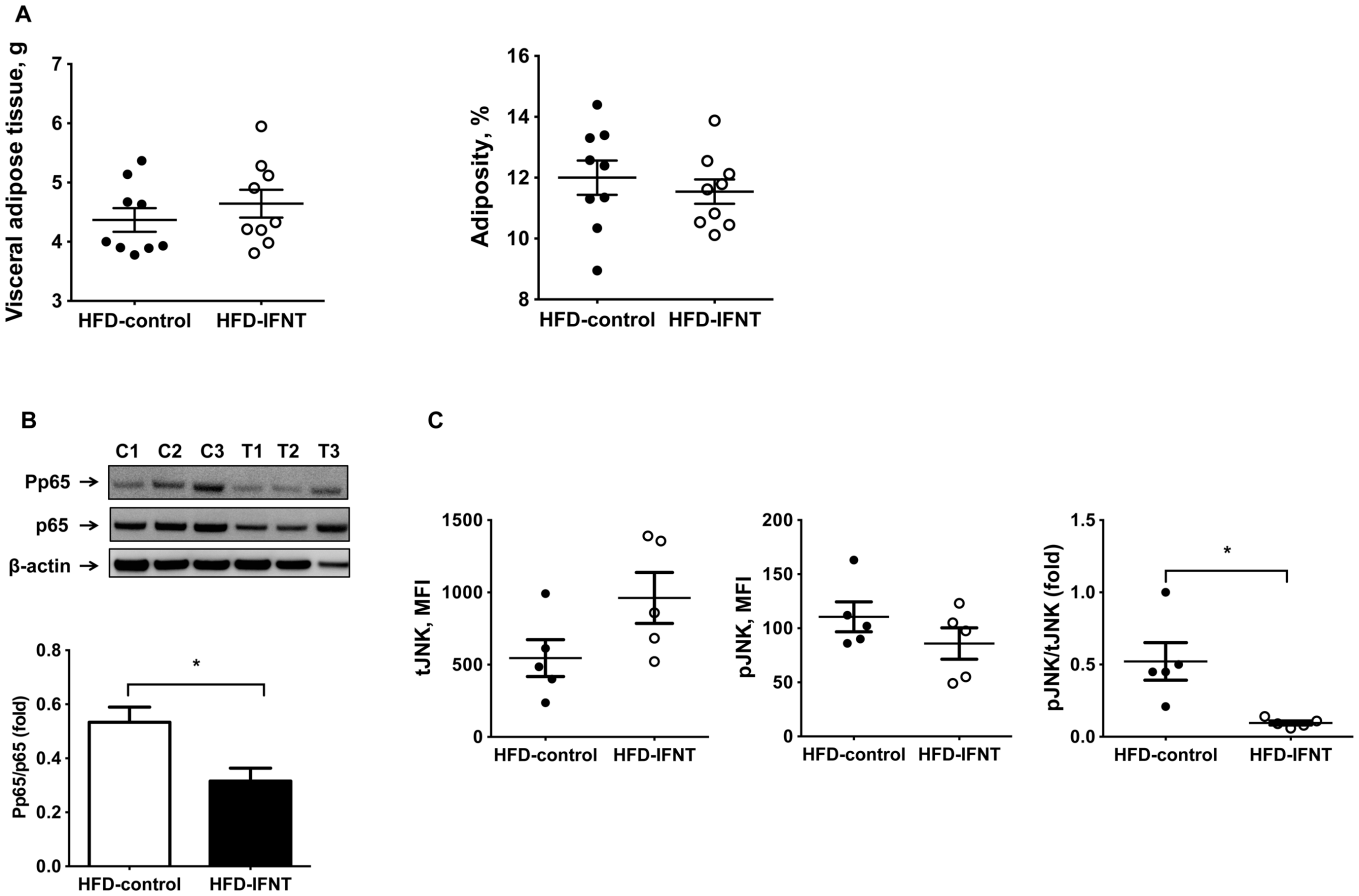
IFNT reduces obesity-associated adipose tissue inflammation. (A) Visceral adipose tissues (VAT) weight and adiposity of mice after 12-week HFD feeding. (B) Nuclear factor-κB (NFκB) activation in VAT of HFD mice. Western blotting was performed with antibodies against p65 and phosphorylated p65 (Pp65; n = 3). C, control; T, IFNT. (C) Activation of c-Jun N-terminal kinase (JNK) signaling pathway in VAT of HFD mice. Fluorescent-labeled beads conjugated with antibodies against total JNK (tJNK) and phosphorylated JNK (phospho JNK) in VAT were measured using the Bio-Plex® MAGPIX™ multiplex reader. MFI, medium fluorescence intensity. Data are presented as mean ± SEM. *P<0.05.

BMDMs were stimulated by lipopolysaccharide (LPS; 100 ng/mL) for M1 activation or IL-4 (20 ng/mL) for M2 activation. To test the dosage effects of IFNT, BMDMs were treated with IFNT at 10,000, 5,000, or 1,000 anti-viral units (AVU)/mL for 48 h with LPS or IL-4 (FigureS2). After 48 h of stimulation, BMDMs were examined for activation of expression of associated surface antigens CD69, CD80, and CD86 using flow cytometry.

### Flow cytometry analysis

Unless specified, antibodies were obtained from eBioscience. Vascular stromal cells (VSC) of VAT and BMDMs were stained with fluorescence-conjugated antibodies to detect cell lineages in VAT or their activation. B cells were detected with antibodies against B220, CD19, CD5 and CD43; T cells were detected with antibodies against CD4 and CD8; macrophage subtypes and activation of macrophages were detected using antibodies against F4/80, CD11b, CD206 (Biolegend), CD11c, CD80, CD86, and CD69. Phosphorylated signal transducers and activators of transcription 1 (STAT1) and STAT3 of BMDMs were detected using an intracellular staining assay. Flow cytometry analysis was performed using Accuri C6 (BD Bioscience), and results were analyzed using Flowjo or Accuri C6 software (BD Bioscience).

### Immunohistochemistry

Tissues collected from HFD-fed mice were fixed andstained with antibodies against F4/80, B220, and CD3 (eBioscience) to detect macrophages, B cells, and T cells, respectively. Immunoglobulin (IgG) protein was used as the negative control. Images were captured using a Zeiss Stallion Dual Detector Imaging System with Intelligent Imaging Innovations Software (Carl Zeiss).

### Western blotting

After homogenization of VATs using the BeadBug™microtube homogenizer (Benchmark Scientific),total protein was extracted from VAT homogenate using a RIPA buffer, and protein concentrations were determined using the Bradford assay. Proteins were separated on PROTEAN® TGX Stain-Free™ Precast Gel (Bio-Rad) and transferred onto a polyvinylidene fluoride (PVDF) membrane followed by detection using antibodies directed against the respective antigens. Activation of NFκB pathway in adipose tissues was evaluated using antibodies against p65 and phosphorylated p65 (Pp65; Cell Signaling Technology®).

### Quantitative reverse transcriptase-polymerase chain reaction (qRT-PCR) analysis

Total RNA was extracted from adipose tissues or BMDMs using the Trizol extraction protocol according to the manufacturer's instructions. Gene expression analysis was performed using a iScript One-Step RT-PCR kit with SYBR Green (Bio-Rad) on Bio-Rad CFX384 (Bio-Rad). The data presented correspond to the mean of 2^−ΔΔCt^ from at least three independent experimentsafter being normalized to β-actin.

### Bio-Plex protein expression assay

The concentrations of IL-1β, TNF-α, IL-6, IL-10, and chemokine (C-C motif) ligand 2 (CCL2) in plasma were determined using Bio-Plex™Cytokine Assay (Bio-Rad). Concentrations of insulin in plasma were determined using the Bio-Plex Pro Mouse Diabetes Insulin set (Bio-Rad). The levels of total and phosphorylated JNK, and total and phosphorylated Akt in mature adipocytes were determined using the Bio-Plex Cell Signaling Magnetic Assays (Bio-Rad). These Bio-Plex assays were performed using the Bio-Plex MAGPIX™ multiplex reader (Bio-Rad). Results were analyzed using Bio-Plex Data Pro™ software (Bio-Rad).

### Data and statistical analyses

Results are expressed as means ± SEM. Each data point derived from qRT-PCR assays represents an average of two technical replicates, and data were averaged over independently replicated experiments (n = 3–4 independently collected samples) and analyzed using the Student's*t* test. The overall group-effect was analyzed for significance using two-way ANOVA and Bonferroni post-test for each factor at each individual time. Data analyses were performed using Graphpad Prism version 6.0 software. A value of *P*<0.05 was considered statistically significant.

## Results

### Interferon tau alleviates obesity-induced insulin resistance

To evaluate the effects of IFNT on obesity-associated inflammation and insulin resistance, we adopted a dietary-induced obesity model. An effective IFNT dose was chosen based on previous studies [37]. After 12 weeks feeding, IFNT treatment did not significantly affect body weight gain or food intake in either HFD or LFD groups (Figure 1A, B). However, compared to the control HFD mice (HFD-Control mice), IFNT treatment (HFD-IFNT mice) decreased hyperglycemiaand blood insulin levels (Figure 1C). In addition, HFD-IFNT mice had lower glucose and insulin levels after 16-h of fasting compared to the HFD-Control mice (Figure 1C). To evaluate the effects of IFNT on insulin sensitivity, mice were subjected to glucose and insulin tolerance tests. Mice were fasted for 16 h and then injected with a single dose of glucose (2 mg of glucose per gram of body weight) or insulin (1 U of insulin per kg of body weight) followed by determination of concentrations of blood glucose at various time points (Figure 1D, E). With LFD mice, IFNT treatment did not alter concentrations of glucose or insulin in plasma; whereas HFD-IFNT mice had lower concentrations of glucose than HFD-Control mice (Figure 1D, E). Collectively, these results suggest that IFNT improves obesity-associated glucose metabolismand insulin sensitivity.

### Interferon tau alleviates obesity-associated inflammation

**Figure 3:**
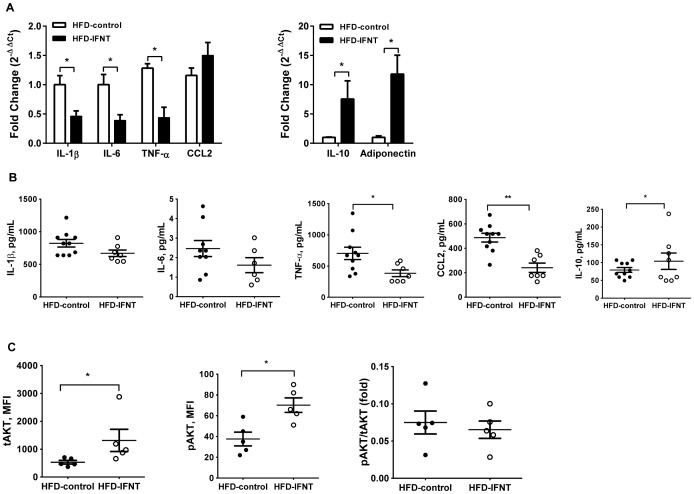
IFNT administration altered the cytokine profile and insulin signaling. (A) Gene expression of cytokines. Cytokines, chemokine (C-C motif) ligand 2 (CCL2) and adipokineadiponectin in VAT of HFD mice measured using quantitative reverse transcriptase-PCR (qRT-PCR; normalized to β-actin). IL, interleukin; TNF-α, tumor necrosis factor-α. (B) Concentration of IL-1β, IL-6, TNF-α, CCL2, and IL-10 in plasma of HFD mice were measured using the Bio-Plex™Cytokine Assay (Bio-Rad). (C) Adipose tissue insulin signaling. After 16-h fasting, mature adipocytes were collected from VAT and treated with insulin (100 nM) for 15 min. Total AKT (tAKT) and phosphorylated AKT (pAKT) protein in adipocytes were measured using Bio-Plex Cell Signaling Magnetic Assays (Bio-Rad). Data are presented as mean ± SEM. *P<0.05.

There is compelling evidencefor causal effects of obesity-associated chronic inflammation, especially in adipose tissues of obese individuals, and the pathogenesis of systemic insulin resistance [1]. Although HFD-IFNT and HFD-Control mice had similar degrees of adiposity (Figure 2A), IFNT treatment suppressed activation of NFκBactivation as evidenced by lower p65 phosphorylation and JNK pathway activity as evidenced by decreased phosphorylated-JNK in VAT of HFD mice (Figure 2B, C). In addition, HFD-IFNT mice displayed lower levels of proinflammatory cytokines, namely IL-1β, IL-6 and TNF-α, and increased expression of the anti-inflammatory cytokine IL-10 in adipose tissuecompared to HFD-Control mice (Figure 3A). However, the expressionof CCL-2 in adipose tissues isolated from HFD mice was not affected by IFNT treatment (Figure 3A). Expression of adiponectin, an adipokine negatively associated with obesity, was induced by IFNT treatment in HFD mice compared to the control group (Figure3A). We also detected decreasedconcentrations ofCCL2 and TNF-α and elevated concentration of IL-10 in plasma of HFD-IFNT mice compared to HFD-Control mice (Figure 3B). We further determined the impact of IFNT treatment on the insulin signaling pathway in adipose tissues of HFD mice. In the *ex vivo*analysis of insulin signaling, we detected an increase in the abundance of phosphorylated Akt protein in isolated mature adipocytes treated with insulin compared to that in mature adipocytes from mice that were not treated with insulin. This result indicates that insulin successfully activated the intracellular insulin signaling pathway in the isolated mature adipocytes.Interestingly, total Akt protein and its insulin-responsive phosphorylation were both greater in mature adipocytes isolated from HFD-IFNT mice than HFD-Control mice (Figure 3C).Taken together, our results suggest that IFNT treatment effectively modulates obesity-associated insulin resistance at least by suppressing tissue inflammation.

### Interferon tau regulates macrophage activation in adipose tissues of HFD mice

**Figure 4:**
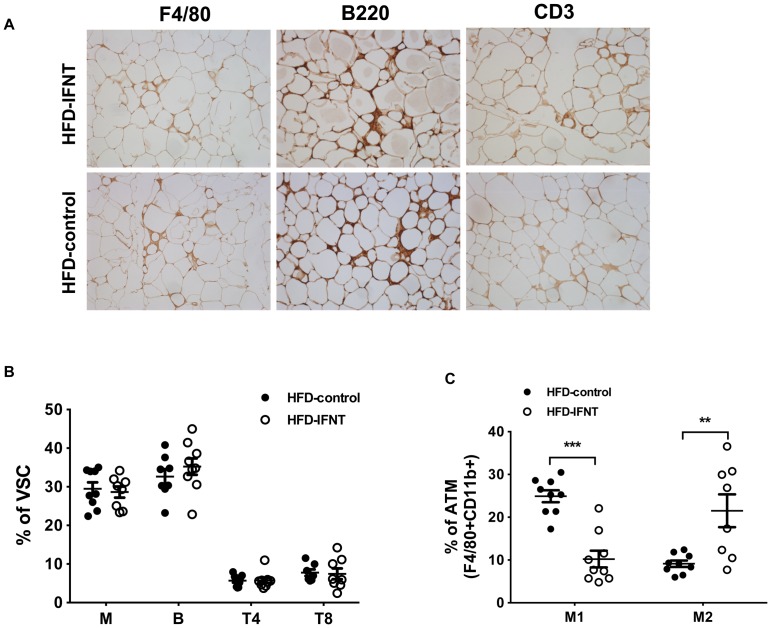
IFNT regulates macrophage activation in adipose tissues of mice fed a HFD. (A) Adipose tissue sections of HFD mice were stained with antibodies against F4/80, B220 and CD3 for macrophages, B cells and T cells, respectively. (B) Macrophage (M), B cell (B), CD4^+^ T cell (T4) and CD8^+^ T cell (T8) infiltration in VAT of HFD-fed mice was analyzed by flow cytometry with antibodies against F4/80, CD11b, B220, CD4 and CD8. (C) Macrophage subtypes in visceral fat stromal cells (VSC) of VAT were analyzed by flow cytometry using antibodies against F4/80, CD11b, CD11c and CD206. Data are presented as mean ± SEM. *P<0.05, **P<0.001, ***P<0.0001.

To further understand the impact of IFNT on adipose tissue immune cell populations that are major contributors to adipose tissue inflammatory status [4]-[6], [13], we examined the relative proportions of T cells, B cells and macrophages in visceral stromal cells of VAT from HFD-IFNT and HFD-Control mice. We first performed immunohistochemical staining on visceral adipose tissues isolated from both HFD-IFNT and HFD-Control groups with antibodies against F4/80 (macrophages), B220 (B cells) and CD3 (T cells). The results indicated that total numbers of macrophages, B cells and T cells infiltrated into VAT are comparable in HFD-IFNT mice and HFD-Control mice (Figure 4A). This was confirmed by further analysis using flow cytometry assays with the same set of antibodies. Consistent with results of immunohistochemical staining, the proportions of total macrophages (F4/80^+^CD11b^+^), B cells (B220^+^) and T cells (CD4^+^ or CD8^+^) were not significantly different between IFNT treated and control mice on either HFD (Figure4B) and LFD (FigureS3). Surprisingly, the distribution of macrophage subpopulations, i.e. M1 and M2 macrophages, was significantly altered by IFNT treatment. Compared to the HFD-Control mice, HFD-IFNT mice displayed dramatically decreasedproinflammatory M1 macrophages (F4/80^+^CD11b^+^CD206^-^CD11c^+^; Figure 4C)and a significant increase in anti-inflammatory M2 macrophages (F4/80^+^CD11b^+^CD206^+^CD11c^-^; Figure4C) which suggests a regulatory role for IFNT in macrophage polarization.

### Interferon tau modulates macrophage polarization

**Figure 5:**
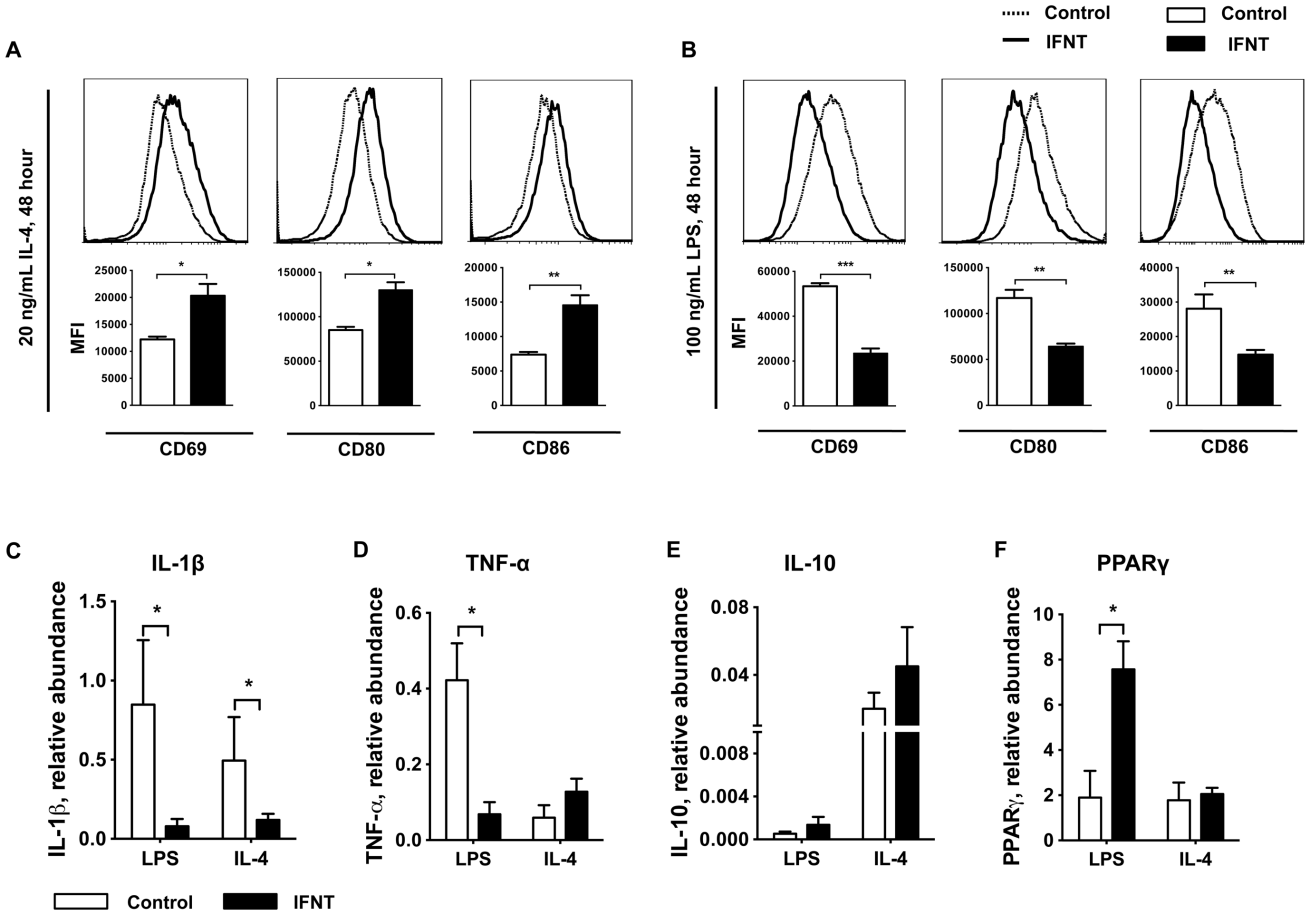
IFNT modulates macrophage polarization and cytokine profiles. The surface makers CD69, CD80, and CD86 of BMDMs were analyzed using flow cytometry after 48-hIL-4 (20 ng/mL; A) or lipopolysaccharide (LPS, 100 ng/mL; B) stimulation (n = 3). BMDMs were treated with IFNT at 5,000 antiviral units (AVU)/mL. The expression of cytokines IL-1β (C), TNF-α (D) and IL-10 (E) and peroxisome proliferator-activated receptor γ (PPARγ) (F) in BMDMs activated in the presence of IFNT (black bars) were analyzed by qRT-PCR (normalized to β-actin, n = 3) and compared to activated BMDMs with no IFNT treatment (white bars). Data are presented as mean ± SEM. *P<0.05, **P<0.001, ***P<0.0001. MFI, medium fluorescence intensity.

Given the distinct shift in activation status of adipose tissue macrophages *in vivo*, we further evaluated the effects of IFNT on macrophage polarization using a well-established *in vitro* model [36].Bone marrow-derived macrophages were treated with IFNT at various dosage in the presence of LPS (100 ng/mL) for M1 activation or IL-4 (20 ng/mL) for M2 activation and activation of associated surface antigens was determined using flow cytometry assays. M2 macrophages (IL-4 treatment) displayed a significantly enhanced activation pattern as judged by stronger induction of surface markers CD69, CD80, and CD86 at 48 h after stimulation (Figure 5A). In contrast, activation of M1 macrophages induced by LPS treatment was significantly stalled in the presence of IFNT resulting in the left shift of surface marker levels (Figure 5B). We further examined the cytokine production profiles in these M1 and M2 macrophages using qRT-PCRanalysis. As expected, IFNTsignificantly suppressed expression of proinflammatory cytokines IL-1β and TNF-α by BMDMs in response to LPS stimulation compared to control BMDMs (Figure5C, D). In addition, cells also displayed a slight increase in IL-10 upon IL-4 stimulation in the presence of IFNT (Figure5E). PPARγ is a key regulator that suppresses proinflammatory M1 and promotes anti-inflammatory M2 activation. Interestingly, IFNT did not affect IL-4-dependent PPARγexpression in M2 macrophages, but IFNT significantly increased PPARγ expressionin M1 macrophages (Figure 5F), which suggests a potent inflammatory suppressing impact of IFNT on macrophage polarization.

**Figure 6:**
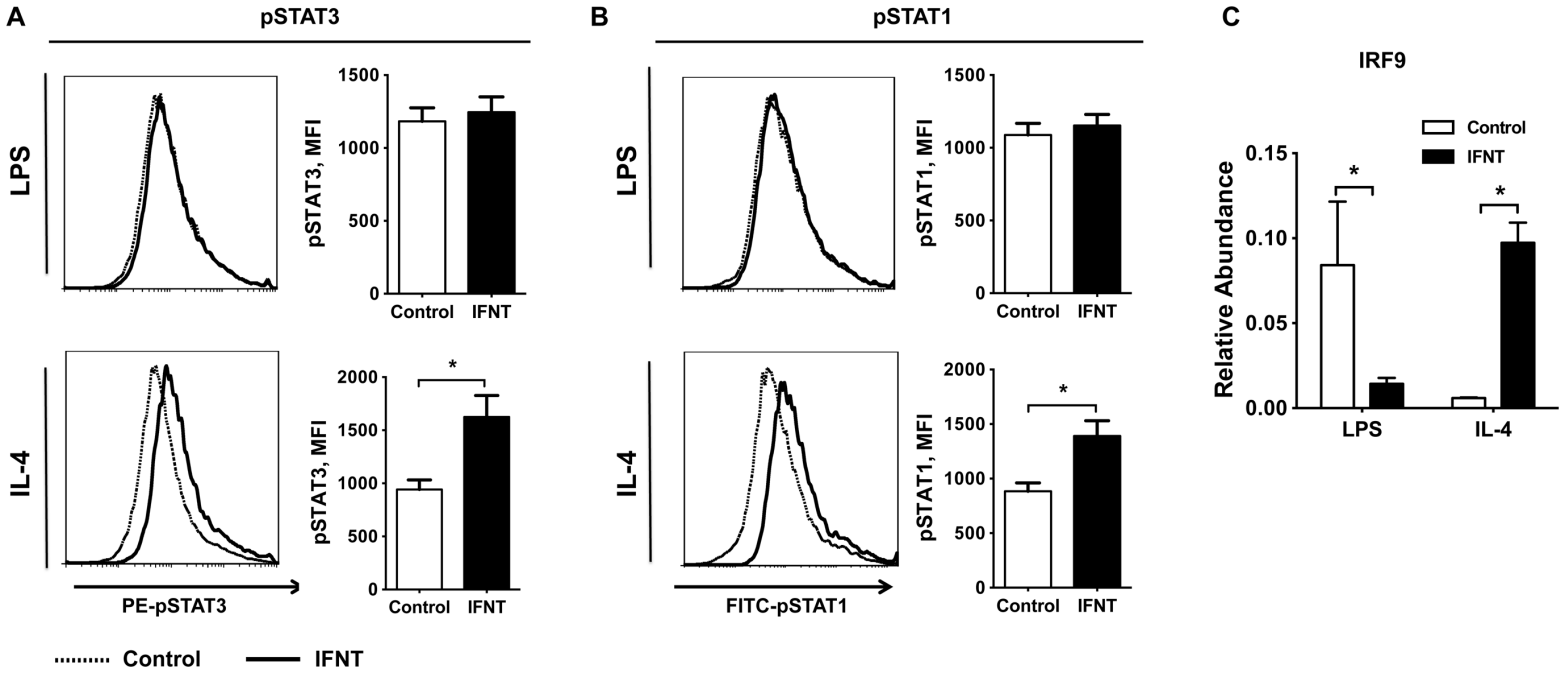
IFNT induces STAT1 and STAT3 activation in BMDMs. (A) The phosphorylation of signal transducer and activator of transcription 3 (pSTAT3) and (B) pSTAT1 in BMDMs was measured by flow cytometry after 90-min of LPS (100 ng/mL) or IL-4 (20 ng/mL) stimulation (n = 3). (C) The expression of interferon regulatory factor 9 (IRF9) was analyzed by qRT-PCR after 48-h LPS (100 ng/mL) or IL4 stimulation (20 ng/mL); n = 3. Data are presented as mean ± SEM. *P<0.05, **P<0.001.

To understand the mechanism of IFNT action in regulating macrophage polarization, we examined signaling pathways mediated by type I interferon receptors in activated macrophages. We found that the expression of type I interferon receptor (IFNAR) was not affected by IFNT treatment in activated BMDMs (FigureS4). However, IFNT significantly enhanced STAT3 activation upon IL-4 stimulation as evidenced by significantly elevated phosphorylated STAT3 proteindetected by intracellular staining assays followed by flow cytometry analysis (Figure6A). Alternatively, type I interferons can exert anti-inflammatory functions through inducing activation of interferon-stimulated gene factor-3 (ISGF3) complex, which includes STAT1, STAT2, and interferon regulatory factor 9 (IRF9). In M2 macrophages, activation of STAT1 was increased by IFNT compared to the control (Figure6B).In LPS-stimulated BMDMs, IFNT did not affect the phosphorylation status of STAT1 or STAT3 (Figure6A, B). In addition, the abundance of IRF9 was significantly decreased in M1 macrophages, but increased in M2 macrophages in response to IFNT (Figure6C). Thus, these results suggest that IFNT may modulate macrophage polarization primarily through controlling activation of ISGF3 complex and STAT3 pathway.

## Discussion

Adipose tissue inflammation is a major contributor to the pathogenesis of obesity-associated insulin resistance [1]–[7].Using a diet-induced obesity model, we observed a significant increase in inflammation at both adipose tissue and systemic circulation levels in obese mice, accompanied by exacerbated insulin resistance. This is consistent with previous reports and confirms the direct correlation of tissue inflammation with obesity-associated insulin resistance [1]–[7]. Thus, our research provided a new set of evidences to support the development of therapeutic strategy with anti-inflammatory agents to treat patients with obesity-induced insulin resistance and subsequent type 2 diabetes. Type I interferons exert profound anti-inflammatory effects through modulating immune cell functions [24]. However, patients treated with IFNA and IFNB, two of the available options in this category, often display severe high fever, as well as damage to liver and kidney functions [27], [28], [38].Such severe adverse effects greatly stall the beneficial expectation of their applications. Compared to IFNA and IFNB, IFNT displays similar immunomodulatoryfunctions but low cytotoxicity even at higher dose, thus can provide a new option to treat obesity-induced insulin resistance and autoimmune disorders [31], [33].

Type I interferons share high similarities among their members in both primarynucleotide coding and protein folding structure [30]. Structural analysis demonstrates that IFNT binds to type I interferon receptors to activatetype I interferon intracellular signaling pathways that alter immune status [39].This is confirmed in our study using an *in vitro* culture system. IFNT-treated BMDMs displayed a significantly suppressed inflammatory response to LPSaccompanied by decreased production of IL-1β and TNF-α. This effect is partially mediated by suppressingthe ISGF3 pathway. In addition, IFNT significantly enhanced the anti-inflammatory response, namely activation of M2 macrophages. Accordingly, decreased expression of IL-1β was observed in BMDMs stimulated with IL-4 in the presence of IFNT. Our *in vivo* results further confirmed the anti-inflammatory effects of IFNT in adipose tissues, evidenced by suppression on inflammatory pathwaysand production of proinflammatory cytokines.

Given the potent anti-inflammatory effects of IFNT *in vivo*, it is not surprising that IFNT ameliorated insulin resistance in the obese mice. IFNT administration did not affect body weight gain or adiposity of obese mice, or metabolic status of adipose tissues and liver of obese mice (Figure S5), but there was a decrease in concentration of triglycerides in plasma from HFD-IFNT mice (Figure S6). These results are not consistent with results of a previous study withZucker diabetic fatty rats [37].This may be due to differences in animal models and length of IFNT treatment of the rats to 12 weeks of age. Intriguingly, the composition of infiltrated immune cells, especially the macrophage subtypes, in the adipose tissueswas significantly altered upon IFNT treatment. The overall adipose tissue immune cells including T cell, B cell and macrophage populations in the mice treated with IFNT were comparable to the control mice, suggesting that IFNT treatment did not affect immune cell recruitment, which was concomitant with unchanged expression of CCL2in adipose tissuesfrom IFNT-treated HFD mice and control mice. However, adipose tissue inflammation was significantly suppressed by IFNT treatment as evidenced by decreased proinflammatory cytokine expression levels, which was associated with elevated Akt activation upon insulin stimulation. This is partially attributed to the shift of adipose tissue macrophage status distribution resulting in more M2 than M1 macrophages. The M2 macrophages exert anti-inflammatory effects in the tissue microenvironment including regulation through increasing secretion of anti-inflammatory cytokines, including IL-10 [13]. We observed greater abundance of IL-10 in HFD mice treated with IFNT. Results of a previous study suggest that type I interferons act through either the ISGF3 complexity or STAT3 in immune cells [24], [25]. Our results suggested that IFNT may function through the ISGF3 complexin macrophages and also exerts a significant impact on STAT3 phosphorylation, resulting in significantly enhanced production of IL-10. Further understanding the mechanism of IFNT action in mitigating obesity-associated symptoms and its potential impact on other immune cell activation await further investigations.

In summary, results of our study demonstrate that IFNT is a potent regulator for obesity-associated insulin resistance and tissue inflammation that is accounted for partially by effects of IFNT to control adipose tissue macrophage polarization. Given its low cytotoxicity compared to other members of the type I interferon family, our study provides the first evidence to support the potential application of IFNT to mitigate obesity-associated syndromes including various autoimmune diseases.

## Supporting Information

Figure S1 **Concentrations of interferon tau (IFNT) in plasma of HFD-fed mice were determine by radioimmunoassay (n = 4–5).** Data are presented as mean ± SEM.
(TIF)

Figure S2 **Dosage effect of IFNT on macrophage polarization.** Bone marrow-derived macrophages (BMDMs) were stimulated with LPS (100 ng/mL) or IL4 (20 ng/mL). In addition, BMDMs were treated with IFNT at 1,000(T1), 5,000(T2), 10,000(T3), or 0 anti-viral unit (AVU)/mL (n = 3). After 48 hours, the surface makers CD69, CD80, and CD86 of BMDMs by were analyzed using flow cytometry. Data are presented as mean ± SEM. *P<0.05, **P<0.001.
(TIF)

Figure S3 **The infiltration of immune cells in adipose tissues of LFD mice.** A: Macrophage (M), B cells (B), CD4^+^ T cells (T4) and CD8^+^ T cells (T8) in visceral fat stromal cells (VSC) of visceral adipose tissues (VATs) of LFD mice were analyzed by flow cytometry using antibodies against F4/80, CD11b, B220, CD4 and CD8 (n = 3–4). B: Macrophage subtypes in VSC of VATs of LFD mice were analyzed by flow cytometry using antibodies against F4/80, CD11b, CD11c and CD206 (n = 3–4). Data are presented as mean ± SEM.
(TIF)

Figure S4 **Effect of IFNT on the expression of type I interferon receptor in BMDMs.** The abundance of type I interferon receptor in BMDMs was analyzed by qRT-PCR after 48-hours stimulation with LPS (100 ng/mL) or IL4 (20 ng/mL; n = 3). For the IFNT treatment, BMDMs were treated with IFNT at 5,000 AVU/mL. Data are presented as mean ± SEM.
(TIF)

Figure S5 **The expression of key regulators for lipogenesis, mitochondrial function, lipolysis was measured in the adipose tissues (A) and liver (B) collected from HFD mice or HFD-IFNT mice using qRT-PCR (normalized to β-actin).** Data are means ± SEM, n = 3. ACC, acetyl-CoA carboxylase; FAS, fatty acid synthetase; SCD1, stearoyl-CoA desaturase-1; PGC1β, peroxisome proliferator-activated receptor gamma, coactivator 1 beta; CPT1, carnitinepalmitoyltransferase 1; HSL, hormone-sensitive lipase; G6pase, glucose 6-phosphatase; PEPCK, phosphoenolpyruvatecarboxykinase.
(TIF)

Figure S6 **Concentration of triglyceride in plasma of control or IFNT-treated mice fed a HFD diet or fasted for 16 h.** Data are presented as mean ± SEM.
(TIF)
