# Supplementary figures and images for: Interferon Tau Alleviates Obesity-Induced Adipose Tissue Inflammation and Insulin Resistance by Regulating Macrophage Polarization
Source: PLoS One. 2014 Jun 6;9(6):e98835. doi: 10.1371/journal.pone.0098835 (PMC4048269; doi:10.1371/journal.pone.0098835)

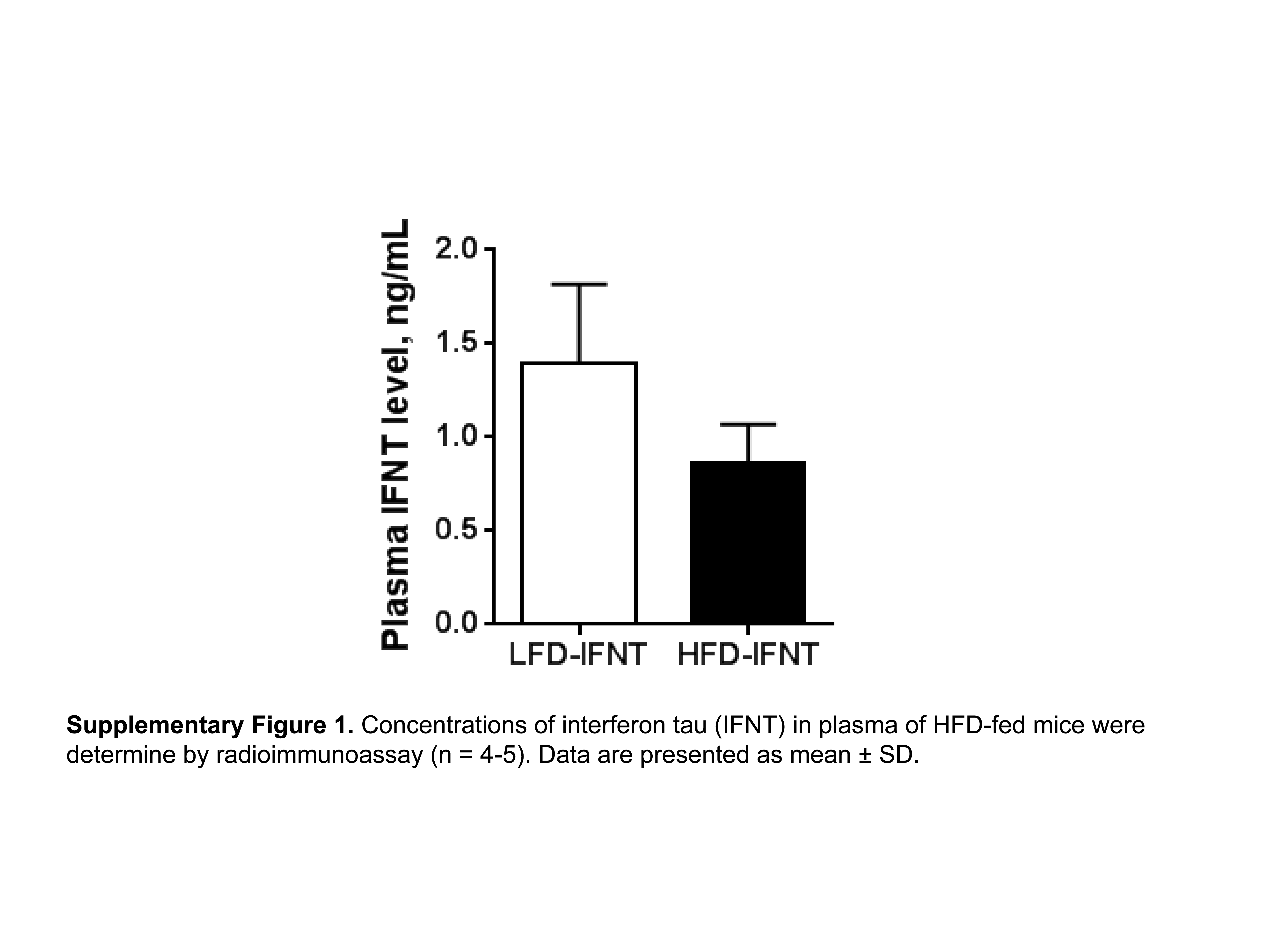

Supplement: Figure S1 — Concentrations of interferon tau (IFNT) in plasma of HFD-fed mice were determine by radioimmunoassay (n = 4–5). Data are presented as mean ± SEM. (TIF) [file pone.0098835.s001.tif]

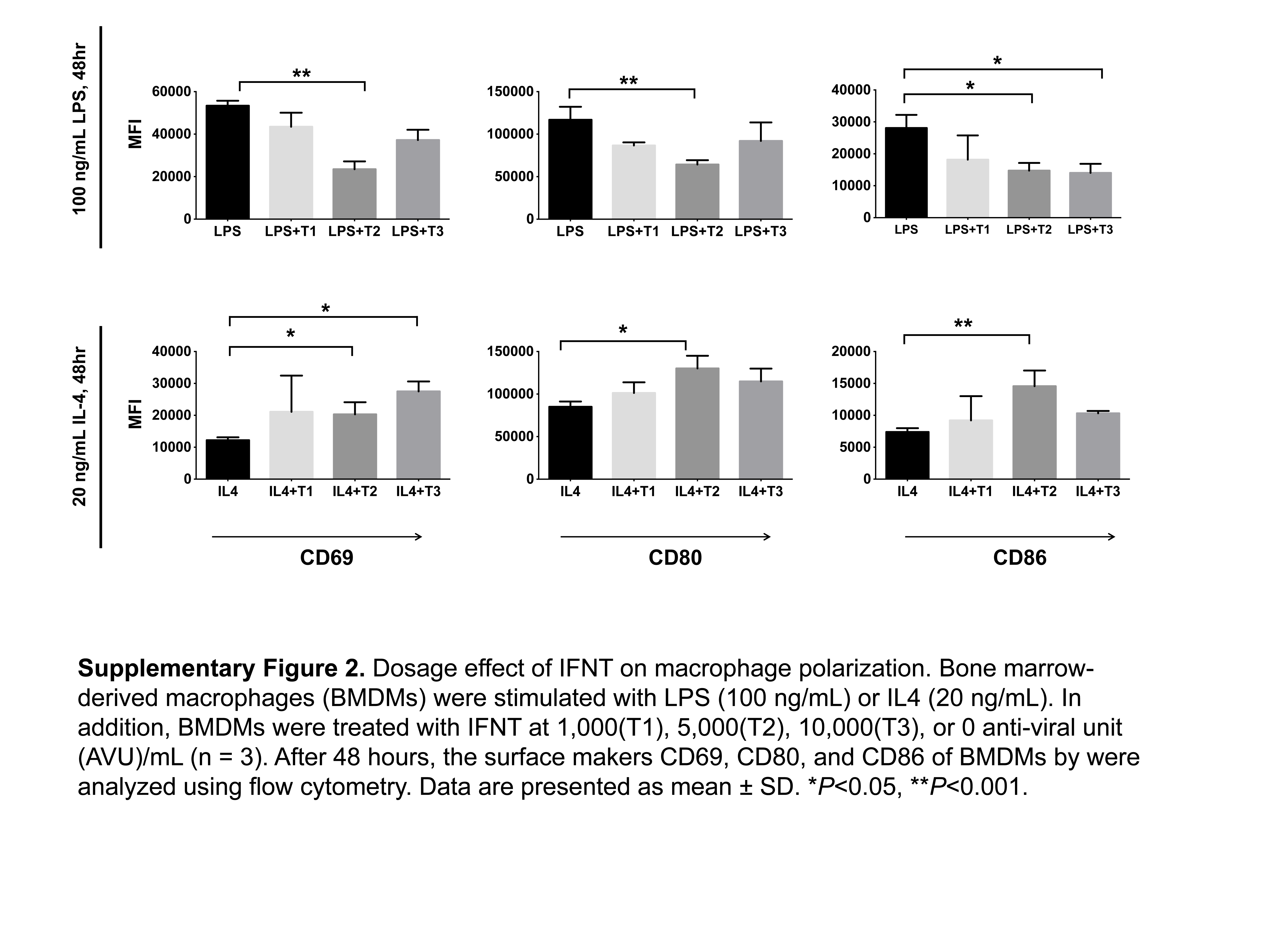

Supplement: Figure S2 — Dosage effect of IFNT on macrophage polarization. Bone marrow-derived macrophages (BMDMs) were stimulated with LPS (100 ng/mL) or IL4 (20 ng/mL). In addition, BMDMs were treated with IFNT at 1,000(T1), 5,000(T2), 10,000(T3), or 0 anti-viral unit (AVU)/mL (n = 3). After 48 hours, the surface makers CD69, CD80, and CD86 of BMDMs by were analyzed using flow cytometry. Data are presented as mean ± SEM. *P<0.05, **P<0.001. (TIF) [file pone.0098835.s002.tif]

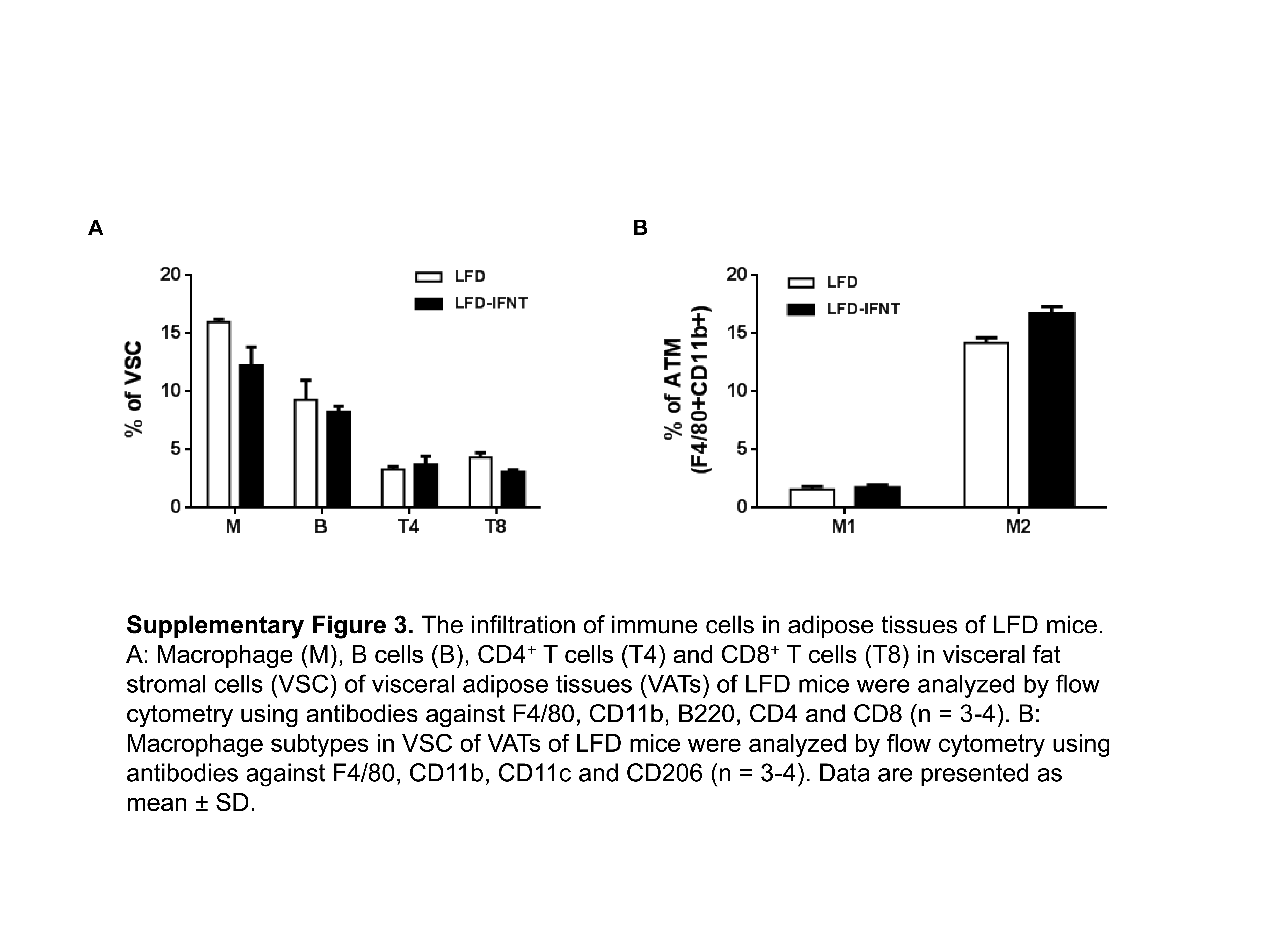

Supplement: Figure S3 — The infiltration of immune cells in adipose tissues of LFD mice. A: Macrophage (M), B cells (B), CD4+ T cells (T4) and CD8+ T cells (T8) in visceral fat stromal cells (VSC) of visceral adipose tissues (VATs) of LFD mice were analyzed by flow cytometry using antibodies against F4/80, CD11b, B220, CD4 and CD8 (n = 3–4). B: Macrophage subtypes in VSC of VATs of LFD mice were analyzed by flow cytometry using antibodies against F4/80, CD11b, CD11c and CD206 (n = 3–4). Data are presented as mean ± SEM. (TIF) [file pone.0098835.s003.tif]

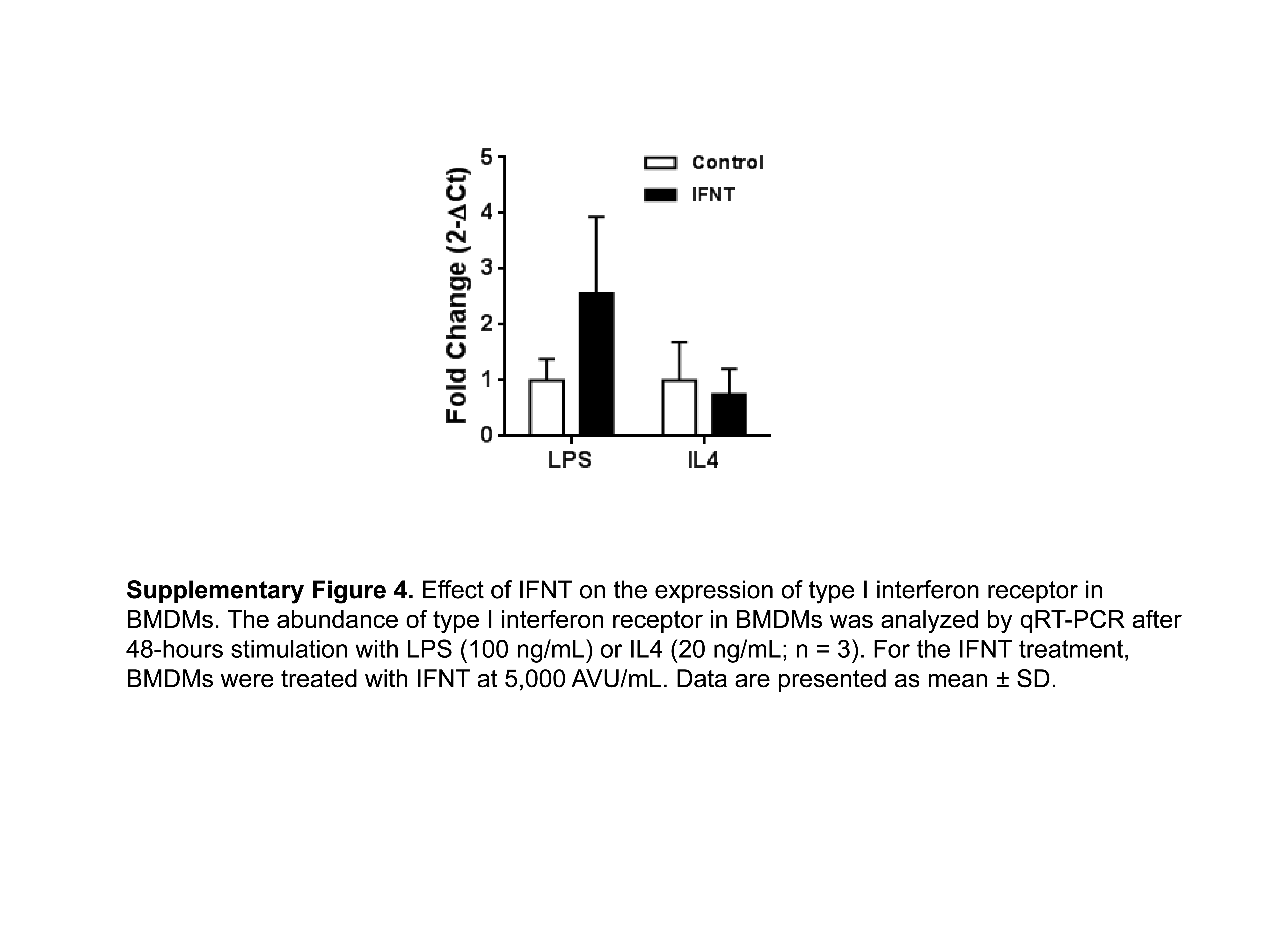

Supplement: Figure S4 — Effect of IFNT on the expression of type I interferon receptor in BMDMs. The abundance of type I interferon receptor in BMDMs was analyzed by qRT-PCR after 48-hours stimulation with LPS (100 ng/mL) or IL4 (20 ng/mL; n = 3). For the IFNT treatment, BMDMs were treated with IFNT at 5,000 AVU/mL. Data are presented as mean ± SEM. (TIF) [file pone.0098835.s004.tif]

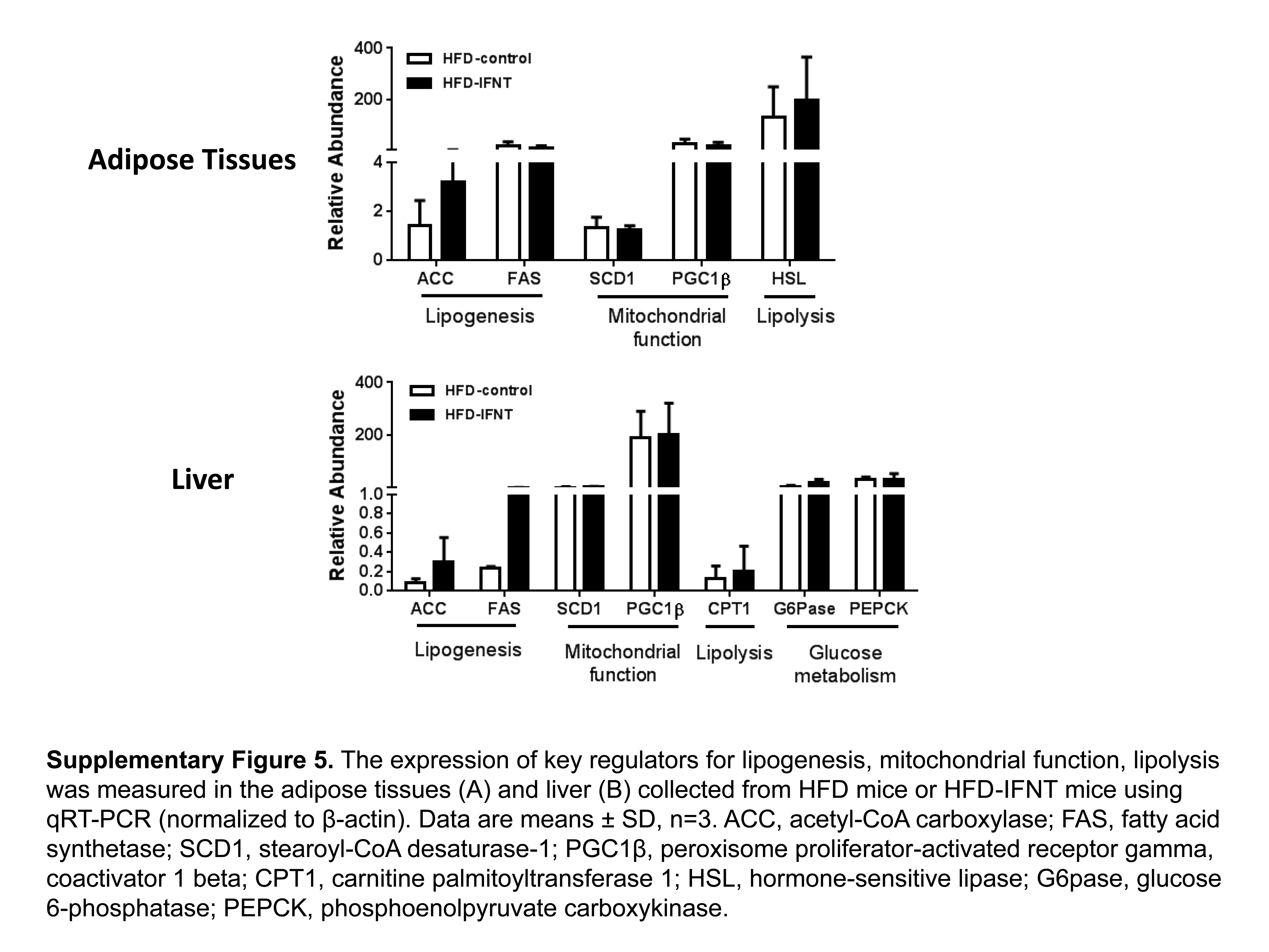

Supplement: Figure S5 — The expression of key regulators for lipogenesis, mitochondrial function, lipolysis was measured in the adipose tissues (A) and liver (B) collected from HFD mice or HFD-IFNT mice using qRT-PCR (normalized to β-actin). Data are means ± SEM, n = 3. ACC, acetyl-CoA carboxylase; FAS, fatty acid synthetase; SCD1, stearoyl-CoA desaturase-1; PGC1β, peroxisome proliferator-activated receptor gamma, coactivator 1 beta; CPT1, carnitinepalmitoyltransferase 1; HSL, hormone-sensitive lipase; G6pase, glucose 6-phosphatase; PEPCK, phosphoenolpyruvatecarboxykinase. (TIF) [file pone.0098835.s005.tif]

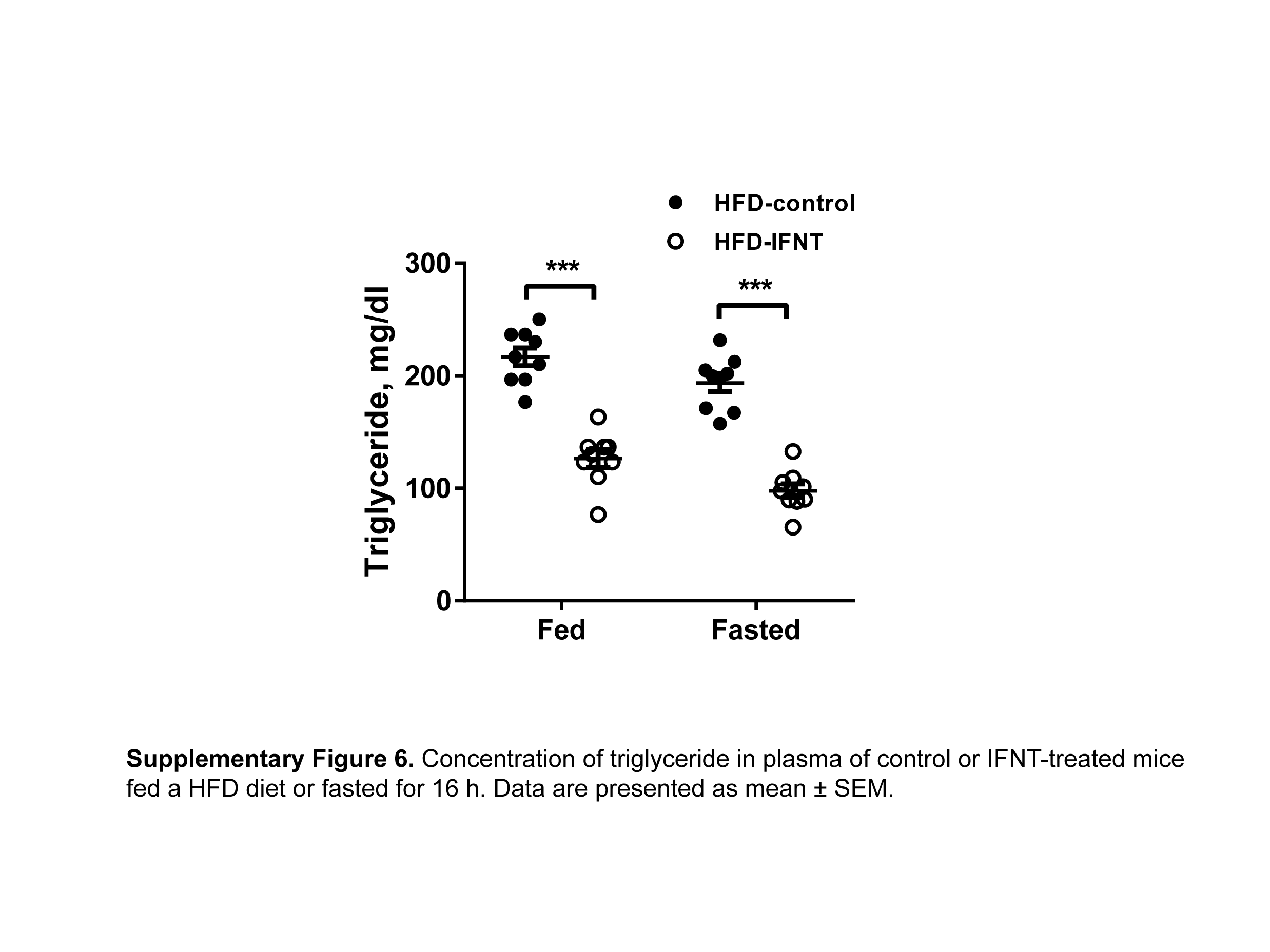

Supplement: Figure S6 — Concentration of triglyceride in plasma of control or IFNT-treated mice fed a HFD diet or fasted for 16 h. Data are presented as mean ± SEM. (TIF) [file pone.0098835.s006.tif]
